# Confocal microscopy analysis reveals that only a small proportion of extracellular vesicles are successfully labelled with commonly utilised staining methods

**DOI:** 10.1038/s41598-021-04225-4

**Published:** 2022-01-07

**Authors:** Genevieve E. Melling, Ross Conlon, Paschalia Pantazi, Elizabeth R. Dellar, Priya Samuel, Luis Alberto Baena-Lopez, Jeremy C. Simpson, David R. F. Carter

**Affiliations:** 1 Department of Biological and Medical Sciences, Oxford Brookes University, Gipsy Lane, Oxford, OX3 0BP UK; 2 Institute of Clinical Sciences, School of Biomedical Sciences, College of Medical and Dental Sciences, University of Birmingham, Edgbaston, Birmingham, B15 2TT UK; 3 Cell Screening Laboratory, School of Biology and Environmental Science, University College Dublin, Science Centre West, Belfield, Dublin 4, Ireland; 4 Institute of Reproductive and Developmental Biology, Imperial College London, Hammersmith Campus, London, UK; 5 Sir William Dunn School of Pathology, University of Oxford, South Parks Road, Oxford, OX1 3RE UK; 6 Evox Therapeutics Ltd, Oxford Science Park, Medawar Centre, Robert Robinson Avenue, Oxford, OX4 4HG UK

**Keywords:** Confocal microscopy, Extracellular signalling molecules, Nanoparticles

## Abstract

Assessing genuine extracellular vesicle (EV) uptake is crucial for understanding the functional roles of EVs. This study measured the bona fide labelling of EVs utilising two commonly used fluorescent dyes, PKH26 and C5-maleimide-Alexa633. MCF7 EVs tagged with mEmerald-CD81 were isolated from conditioned media by size exclusion chromatography (SEC) and characterised using Nanoparticle Tracking Analysis (NTA), Transmission Electron Microscopy (TEM), MACsPlex immunocapture assay and immunoblots. These fluorescently tagged EVs were subsequently stained with C5-maleimide-Alexa633 or PKH26, according to published protocols. Colocalisation of dual-labelled EVs was assessed by confocal microscopy and quantified using the Rank-Weighted Colocalisation (RWC) algorithm. We observed strikingly poor colocalisation between mEmerald-CD81-tagged EVs and C5-Maleimide-Alexa633 (5.4% ± 1.8) or PKH26 (4.6% ± 1.6), that remained low even when serum was removed from preparations. Our data confirms previous work showing that some dyes form contaminating aggregates. Furthermore, uptake studies showed that maleimide and mEmerald-CD81-tagged EVs can be often located into non-overlapping subcellular locations. By using common methods to isolate and stain EVs we observed that most EVs remained unstained and most dye signal does not appear to be EV associated. Our work shows that there is an urgent need for optimisation and standardisation in how EV researchers use these tools to assess genuine EV signals.

## Introduction

Extracellular vesicles (EVs) are small lipid bilayer enclosed structures involved in intercellular communication. They are emerging as key players in many physiological and pathological processes including immune modulation^1,2^, metastasis^3,4^ and neurodegeneration^5^. This functional diversity is linked to the EV-mediated transfer of diverse cellular cargoes (including nucleic acids, lipids and proteins) between distant cells^6^. Extracellular vesicles, either modified or innate, hold much diagnostic^7^ and therapeutic promise^8^, therefore truly understanding their uptake and biodistribution is of paramount importance to the EV field.

EV is an encompassing term for vesicles released by a cell through various biogenesis pathways^9^. Exosomes are EVs that are formed as intraluminal vesicles within multivesicular bodies (MVBs) and are released when MVBs merge with the plasma membrane. They can range in size from 30 to 200 nm. Microvesicles are thought to be larger (around 150–1000 nm) and ‘bud’ directly off from the cell plasma membrane, whilst pinching off with cytosolic cargo^6^. Apoptotic bodies are larger vesicles which are formed when a cell is undergoing apoptosis. In addition to the traditional classifications of EVs, there are several non-canonical EV-like structures, including exomeres, and oncosomes, which are also referred to as belonging to the EV ‘family’^10,11^. Exomeres differ from traditional EV structures as they tend to be smaller (< 50 nm) non-membrane bound particles, but have similar roles protein and nucleic acid cargo delivery^12^, whereas oncosomes are larger EVs (1–10 µm) with implications for carcinogenesis and tumour progression^10^. Given the wide range of origins and classifications of EVs, it comes as no surprise that there is an emerging amount of evidence that there are many different EV subpopulations, each with different molecular markers, lipid profiles, intraluminal cargo and distinct functions (as reviewed in^13^). Therefore, isolating, characterising and visualising a heterogeneous mix of EVs comes with challenges.

To investigate the downstream effects of EVs in recipient cells of with different origins (e.g. glial^14^, endothelial^15^, and bone marrow–dendritic cells^16^), many studies have included EV internalisation assays in which the identification of EVs has been made by the labelling of EV-membranes with lipophilic dyes (e.g. C5-Maleimide-Alexa633 or PKH26)^15,17–19^. However recent studies have revealed unanticipated experimental artifacts linked to these dyes, such as macromolecular aggregates of a similar size to EVs^20^ and their potential to leach off and insert into other adjacent membranes^21^. Despite these shortcomings, these dyes remain commonly used, due to their ease of use, rapid labelling protocols, and signal stability. Furthermore, they are often used to demonstrate bona fide EV uptake using diverse imaging techniques^22–25^.

Alternative EV fluorescent labelling strategies have been used to track tissue biodistribution and cellular uptake of EVs. These have included fluorescently tagging cell membranes, using a bright fluorophore (EGFP/ tandem dimer Tomato; tdTomato) fused to a palmitoylation signal^21^; or tagging EV markers, usually tetraspannins, CD9, CD63 or CD81^26,27^, or others, such as syntenin2 in zebrafish^28^, with a bright fluorophore. Some studies have also used pH-sensitive fluorophores, e.g. CD63-pHluorin, which is fluorescent at neutral pH, to observe EVs both in vitro and in vivo^29–31^. Endogenous EVs have been externally tagged using Membright^28,32^, a synthetic dye that can integrate into the lipid membrane of EVs, SYTO® RNASelect, which selectively binds to free luminal RNA, or C5-maleimide AF488/633 (referred to here as maleimide)^19^, which reacts with thiol groups (-SH) on EV-associated proteins. Previously, maleimide has been shown to be a successful EV label and a good alternative to lipophilic dyes^19^. As the field is still relatively nascent, many of these labelling techniques require further optimisation on each specific experimental scenario.

This study aimed to assess the important question of what proportion of EVs are labelled with commonly used dyes. Here, we combine two methods of fluorescently labelling EVs, by isolating EVs from MCF7 cells expressing an mEmerald fluorescent protein fused to CD81 (mEmerald-CD81) and dual staining with PKH26 or maleimide to assess the efficiency of the labelling techniques. We selected the CD81 construct as it is highly expressed in wild type MCF7 EVs, and is a well characterised EV marker. Surprisingly, we observed that many fluorescently tagged EVs remained unstained and a significant proportion of the dye was not associated with EVs. These unexpected findings caution against interpreting the results using these dyes without a previous optimisation and standardisation process. Our workflow and methodology also constitute a benchmark for assessing the effectiveness of other EV labelling methods.

## Results

### EVs were isolated from MCF7 cells stably overexpressing mEmerald-CD81

**Figure 1:**
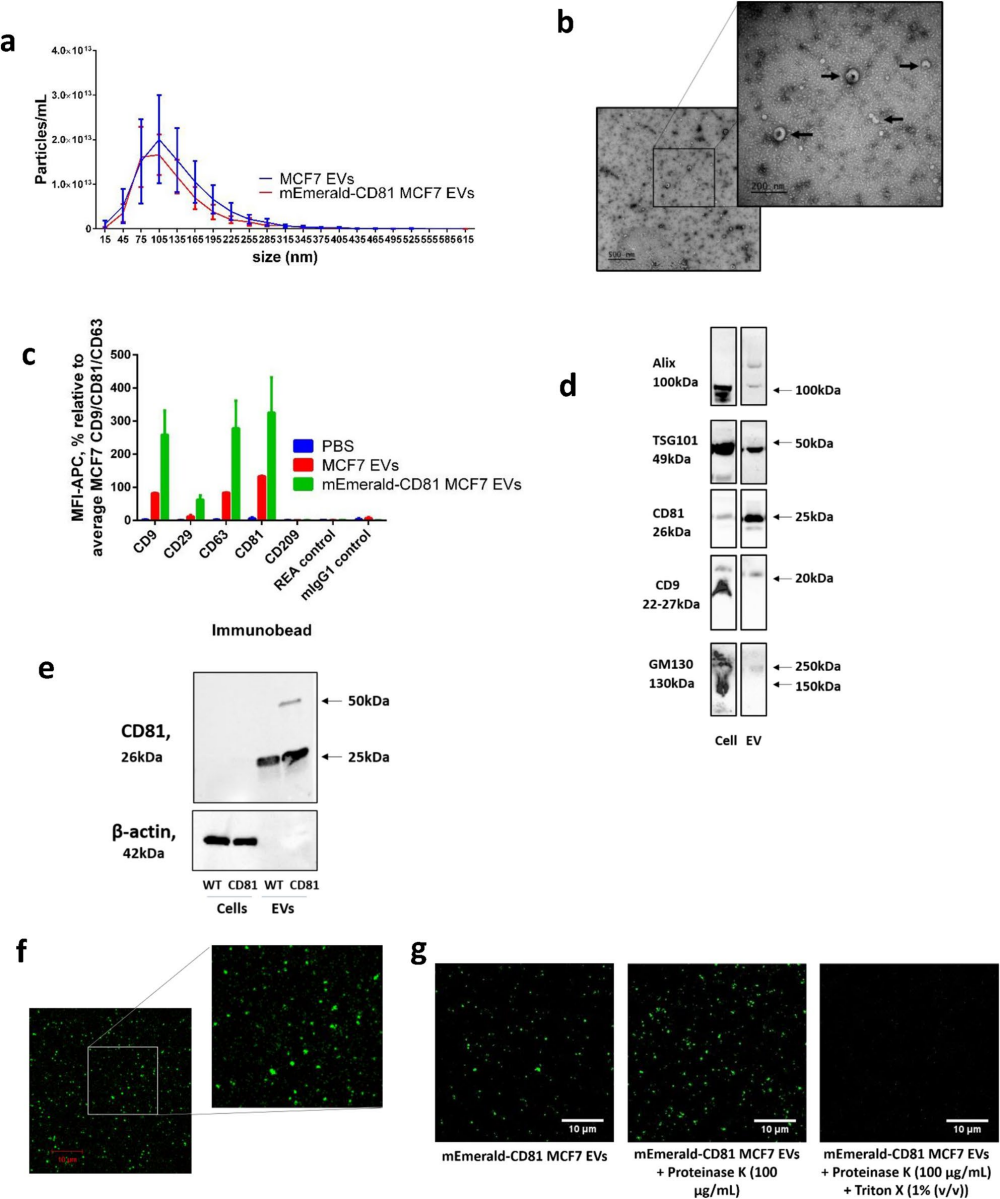
Characterisation of fluorescent mEmerald-CD81 MCF7 EVs. (**a**) Nanoparticle tracking analysis (NTA) was performed on MCF7/ mEmerald-CD81 MCF7 EVs. The graph shows the size distribution of the EVs. N = 5 (biological), error bars = SEM. (**b**) An electron micrograph of negatively stained mEmerald-CD81 MCF7 EVs. Arrows indicate EVs. (**c**) MCF7 and mEmerald-CD81 MCF7 EVs were incubated with MACsPlex immunocapture beads, the beads were then washed and incubated with an antibody cocktail containing APC conjugated antibodies against CD9/CD63/CD81. The histogram shows the APC mean fluorescence intensity for immunobeads CD9, CD29, CD63, CD81, CD209, and negative controls REA and mIgG1. N = 3 (biological), error bars = SEM. (**d**) EV lysate was used in an immunoblot using antibodies for EV positive markers Alix, TSG101, CD81 and CD9, and EV negative marker GM130, with cell lysate as a positive control. Arrows indicate approximate sizes of the bands in kDa. N = 3 (separate batches). (**e**) Lysates from MCF7 or mEmerald-CD81 MCF7s and their EVs were used in immunoblots using antibodies against CD81 and Beta actin. Blots were cropped to make (**d**) and (**e**). (**f**) mEmerald-CD81 MCF7 EVs were placed in 1% (w/v) agarose gel and a coverslip placed on top. (**g**) mEmerald-CD81 MCF7 EVs (left) treated with Proteinase K (100 µg/mL) (middle) or with Proteinase K (100 µg/mL) and Triton X-100 (1% v/v) (right) were placed in 1% (w/v) agarose gel and a coverslip placed on top. Images captured on a Zeiss LSM880 confocal using 100 × objective. N = 3, (biological). Scale bar is 10 µm.

In order to test the staining efficiency of individual EV dyes, we first generated and characterised fluorescently labelled EVs. An MCF7 cell line stably expressing mEmerald-CD81 was created to produce fluorescent EVs. These were then isolated by size exclusion chromatography (SEC) and characterised according to the minimal information for studies of extracellular vesicles (MISEV) guidelines^33^. The size and concentration of EVs were analysed by NTA (Fig. 1a and Table 1), which revealed both mEmerald-CD81 tagged and wildtype EVs to have similar size profiles, with an average size of ca. 100 nm (Fig. 1a). This was consistent with TEM analysis (Fig. 1b), which showed EVs displaying the typical cup shape morphology with a diameter of ca. 100 nm. The presence of classical EV markers was confirmed by a MACsPlex Exosome immunocapture assay (Fig. 1c) and immunoblotting (Fig. 1d). EVs were strongly enriched for tetraspanins CD9, CD63 and CD81 (Fig. 1c). CD29 (Integrin Beta1) was also present on these EVs. However, detection of CD209 (DC-SIGN; Dendritic Cell-Specific Intercellular adhesion molecule-3-Grabbing Non-integrin), a marker specific for macrophages and dendritic cells^34^, and REA and mIgG1 antibody controls was minimal. Immunoblotting confirmed the presence of typical EV markers such as TSG101, Alix, CD9 and CD63, and the absence of GM130, a Golgi matrix protein (Fig. 1d). The fluorescence-tagging was also validated by immunoblotting for the presence of the mEmerald-CD81 (ca. 50 kDa) using a CD81 antibody (Fig. 1e) and by confocal microscopy (Fig. 1f). Triton X-100 and/or Proteinase K treatment of these fluorescent EVs indicated that the fluorescence observed was membrane protected (Fig. 1g), as the signal diminished with Proteinase K and Triton X-100 treatment, but not Proteinase K treatment alone. These results suggested the membrane localisation of our fluorescent signal. Confirming this observation, nanoflow cytometry (NanoFCM) revealed around 55% of all detected particles to be mEmerald-positive in a mEmerald-CD81 MCF7 EV preparation (Supplementary Fig. 1). Taken together, these data confirm the isolation of mEmerald-CD81-labelled EVs.

**Table 1:** Average EV concentration of MCF7 and mEmerald-CD81 MCF7 EV preparations.

EVs	Average Concentration /mL (+/− SEM)
MCF7 EVs	6.05 × 10^11^ ± 2.50 × 10^11^
mEmerald-CD81 MCF7 EVs	1.13 × 10^12^ ± 6.46 × 10^11^

The table shows the average concentration in particles/mL quantified by NTA. N = 5 (biological), error bars = SEM.

### Dyes produce contaminating macromolecular aggregates

**Figure 2:**
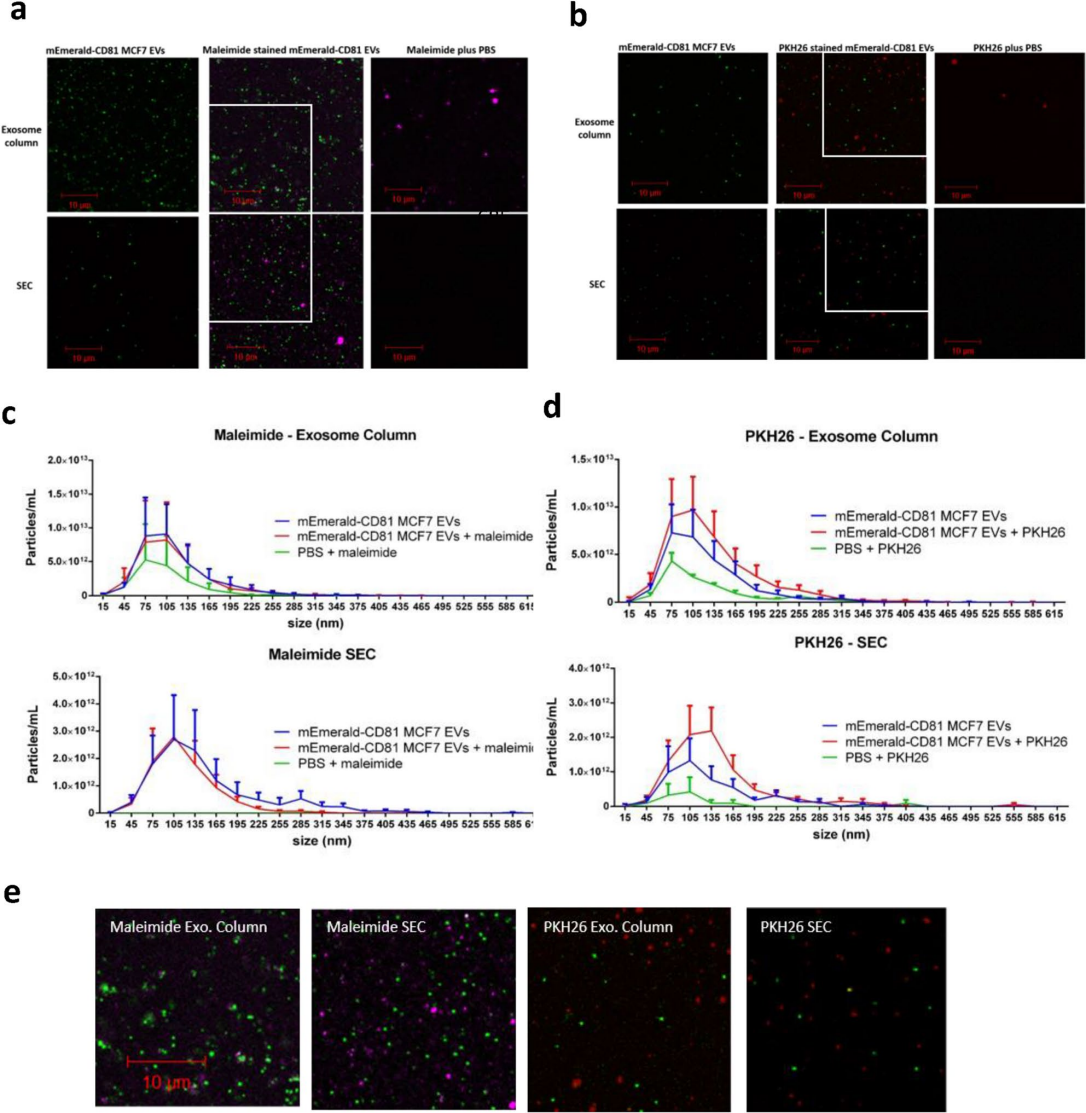
EV stains generate contaminating aggregates. mEmerald-CD81 MCF7s EVs or PBS controls were stained with PKH26 or Maleimide, then excess dye was removed with exosome spin columns (Invitrogen) or a consecutive round of SEC. mEmerald-CD81 MCF7 EVs were stained with maleimide (**a**) or PKH26 (**b**) and stain alone plus PBS (no EVs) were set in 1% (w/v) agarose, a coverslip placed on top and imaged on a Zeiss LSM880 confocal using 100 × objective. Representative images are shown. Particle size and concentration was analysed by NTA for maleimide (**c**) and PKH26 (**d**) stained mEmerald-CD81 MCF7s. A digital zoom. (**e**) is shown for maleimide and PKH26 stained EVs prepared using Exosome columns or SEC as indicated. Scale bar = 10 µm. N = 3 (Biological), error bars = SEM.

Previous studies have demonstrated that certain lipophilic dyes (PKH26 and PKH67) can self-aggregate forming contaminating particles with a similar size to EVs^20,35^. We aimed to assess whether similar aggregation occurs with other EV labels used in the literature. In addition, we tested whether these contaminating dye particulates could be removed. To investigate this, mEmerald-CD81 MCF7 EVs were dual labelled with maleimide or PKH26; subsequently the excess dye was removed using either an Exospin column or an additional round of SEC. The presence of dye aggregates was assessed by NTA and confocal microscopy (Fig. 2). We observed additional particles by both NTA and confocal microscopy in the dye controls (dye plus PBS, with no EVs) for both maleimide (Fig. 2a,c) and PKH26 (Fig. 2b,d) in Exospin column samples. However, when an additional round of SEC was used to remove the dye prior to NTA and confocal analysis, maleimide particles were robustly eliminated; there were no maleimide particles detected by NTA in PBS plus maleimide controls (Fig. 2a and c). With regards to PKH26 particles (plus PBS), after SEC, there was a reduction in the number of aggregates shown by NTA (Fig. 2d), but no particles were observed by confocal analysis (Fig. 2b). In both NTA and confocal analysis, PBS only negative controls showed no visible or quantifiable particles (data not shown). Together, these results confirm previous studies showing that EV-staining dyes can form macromolecular aggregates, and that some techniques may be suitable for reducing their presence.

### EV dyes minimally co-localise with mEmerald-CD81 EVs

**Figure 3:**
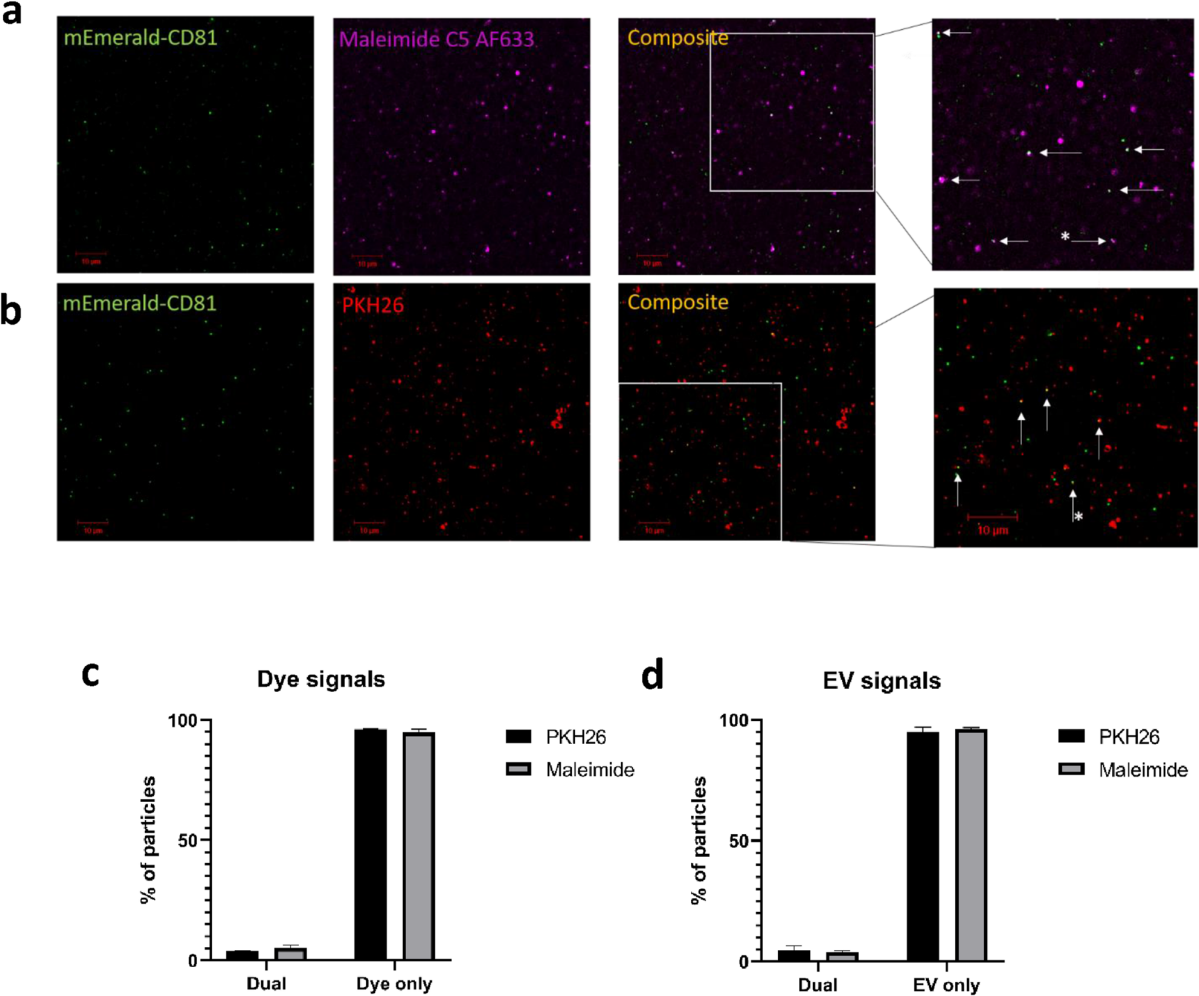
A low percentage of mEmerald-CD81 EVs are dual stained with Maleimide or PKH26. Confocal images of mEmerald-CD81 MCF7 EVs, dual stained with Maleimide C5 AF633 (**a**) or PKH26 (**c**), excess dye removed by SEC and then set in 1% agarose. Green (mEmerald), purple (maleimide C5 AF633; (**a**), and red (PKH26; (**b**) channels are shown along with a composite channel. The composite image has an additional zoomed panel. The white arrows indicate particles where there is colocalisation, the white arrows with an asterisk (*) indicate events where the stain and mEmerald are in close proximity. Scale bar is 10 µm. The fluorescent particle counts were analysed and shown as a % of particles showing the amount of dye that is EV associated (Dual) vs free dye (dye only; **c**). The proportion of mEmerald-CD81 EVs that were unstained (EV only) or dual stained (Dual; EV associated) with either maleimide or PKH26 (**d**). N = 3 (Biological), error bars ± SEM.

Next, we wanted to confirm whether fluorescent EVs were consistently co-stained with each dye. To this end, mEmerald-CD81 EVs were labelled with either maleimide or PKH26, isolated by SEC and set in 1% (w/v) agarose. The confocal inspection of the eluted material showed an extremely and unexpectedly low proportion of co-labelled EVs (Fig. 3a, b). To robustly assess the degree of colocalisation, quantitative analysis was performed using two colocalisation algorithms that work in different ways: the Pearson’s coefficient is widely used to assess colocalisation, but relies solely on correlation of intensity values across an entire image; and rank weighted colocalisation (RWC), that considers both correlating pixels and their relative intensities (Table 2). For maleimide the RWC and Pearson’s values were only 0.11 ± 0.044 and 0.104 ± 0.044 respectively, and for PKH26 the colocalisation values were 0.007 ± 0.008 and 0.0325 ± 0.014, indicating low levels of dual labelling. Fluorescent nanobeads were used to confirm the correct laser alignment of the confocal microscopes (data not shown). These data suggest a poor overlap between the EVs and the dyes used to stain them.

**Table 2:** A table summarising the quantitative colocalisation data of dual labelled mEmerald-CD81 EVs.

Stain used	Pearson’s coefficient	RWC2 (stain overlapping with mEmerald-CD81)	% Dual stained/Colocalisation based on centres of mass-particles coincidence
Maleimide C5 AF633	0.110 ± 0.044	0.104 ± 0.044	5.37% ± 1.77%
PKH26	0.007 ± 0.008	0.033 ± 0.014	4.63% ± 1.61%

Data was acquired from image analysis using the JACOPx ImageJ plugin. N = 3 (biological, 5 technical repeats per biological repeat) ± SEM.

To further characterise the patterns of labelling we quantified the proportions of EV or dye signals that were associated with each other, or were spatially distinct, by using colocalisation based on centres of particle mass coincidence calculation (within the Just another colocalization FIJI plugin). This plugin quantifies individual particles and assesses the overlap of the same pixels on other fluorescence channels, thus calculating the percentage of EV particles that are also positive for EV dye, and therefore assessing the efficiency of the EV staining protocol. In addition, we used this image analysis plugin to quantify the percentage of EV dye particles that were associated with EV particles, this separate analysis indicates the amount of free dye (non EV-associated dye) present in our stained EV preparations. This method showed that only 3.8% of the PKH26 dye overlapped with mEmerald-CD81 particles (Fig. 3c); similarly, only 5.1% of the maleimide signal overlapped with mEmerald-CD81 (Fig. 3c). These data support the evidence presented here and elsewhere that these dyes can form aggregates. Worryingly, further analysis revealed that only 4.8% of the mEmerald-CD81 signals overlapped with the PKH26 signal, while only 3.9% of the EVs overlapped with the maleimide signal (Fig. 3d). These data suggest that the labelling of bona fide EVs is inefficient. Neither Pearson’s correlation, the RWC, nor the percentage of dual labelled EVs appeared to increase following post-labelling clean up with SEC columns compared to exosome columns (Supplementary Fig. 2).

The above data was further supported using Exoview EV characterisation technology (Nanoview). Maleimide stained mEmeraldCD81-MCF7 EVs were used in an Exoview immunocapture assay, where EVs are left to bind to immunocapture spots (CD9, CD63, CD81 and MIgG) and then assessed for size and fluorescence. Our experimental set up allowed for the EVs to be analysed for GFP positivity, maleimide positivity and the presence of CD81+, through the use of a conjugated antiCD81 antibody, enabling us to normalise the data to CD81 + EVs. Interestingly, when we analysed the CD81 + particles bound to the CD81 immunocapture spot, we found that 5.1% of these EVs were positive for both maleimide and CD81- the same percentage colocalisation as found with our previous confocal based calculations (Supplementary Fig. 3). We were also able to assess the colocalisation of maleimide with GFP + particles on CD9 and CD63 immunocapture spots to assess other subpopulations of EVs. We also observed low maleimide +, GFP + positive particles (Supplementary Fig. 4). In addition, by looking at the sizing data, we were able to confirm that maleimide-positive particles were significantly larger than GFP-positive particles, suggesting that maleimide + and CD81 + EVs are largely separate populations (Supplementary Fig. 5 and Supplementary Table 1).

Given the differences in size between maleimide and GFP-positive populations, we next assessed whether the low colocalisation was due to the presence of serum contaminants in our EV preps that were preferentially stained, thus leading to a high proportion of non-EV associated dye. EVs were prepared using serum-free conditioned medium and stained as previously using Exospin columns to remove excess dye. This significantly improved the proportion of dual labelled EVs for PKH26 staining from 4.8% in serum containing conditioned media to 11.0% (p = 0.003), and maleimide staining from 3.9% to 5.6% (p = 0.02; Supplementary Fig. 6 and Supplementary Table 2), compared to serum condition medium extracted EVs prepared in the same way. Taken together these results reveal that EV labelling using these dyes is not specific, and that the majority of the dye is associated with non-vesicular structures that cannot be efficiently removed by the methods tested here.

### Dye and mEmerald-tagged EVs are taken up into cells separately.

**Figure 4:**
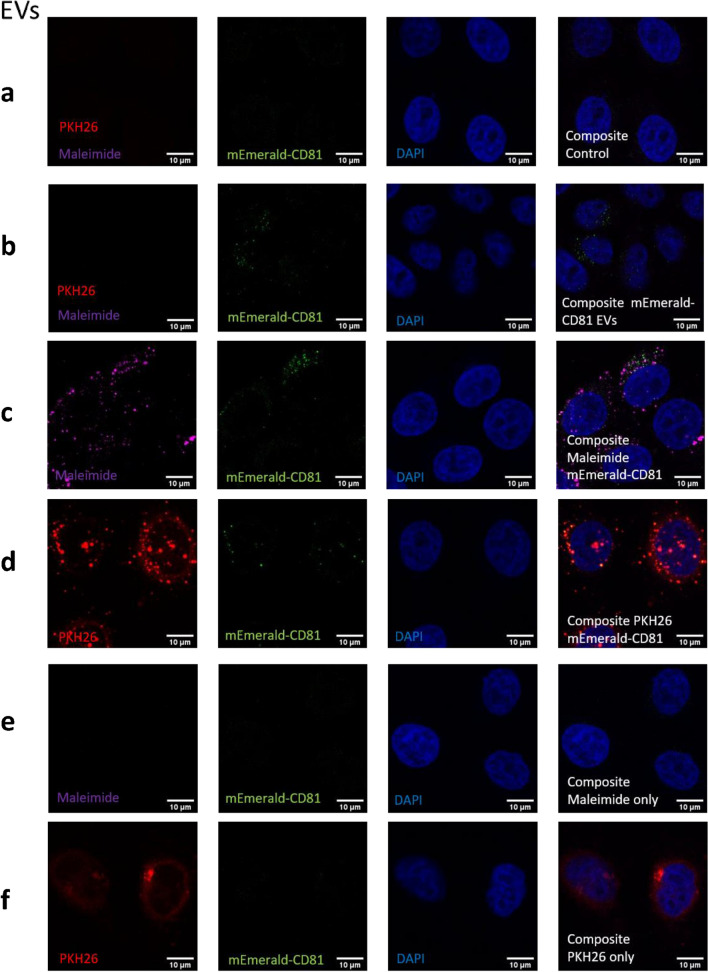
Uptake of dual stained mEmerald-CD81 EVs. Representative confocal images of fixed control (untreated) HeLa cells (**a**) or HeLa cells treated with control (unstained) mEmerald-CD81 EVs (**b**), maleimide C5 AF633 (**c**) or PKH26 (**d**) dual stained EVs, maleimide only (**e**) or PKH26 only (**f**) for 3 h. Scale bars are 10 µm.

Next, we assessed the uptake patterns of maleimide and PKH26 stained EVs and whether each dye was internalised together or separately (Fig. 4a–f). To confirm internalisation into cells, confocal Z-stacks were acquired (data not shown). EVs were stained as before, and to limit the loss of stained EVs for uptake experiments we used exosome columns to remove the excess dye from preparations. For maleimide stained mEmerald-CD81 EVs, although we observed some colocalisation (Pearson’s coefficient 0.212 ± 0.0269; RWC1 (assessing mEmerald-CD81 overlapping with stain) 0.096 ± 0.03; RWC2 (assessing stain overlapping with mEmerald-CD81) 0.261 ± 0.06; Fig. 4c and Table 3) within HeLa recipients at 3 h, the majority of mEmerald-CD81 and maleimide signals appeared to be spatially distinct. With PKH-labelled vesicles the overlap appeared to be higher (Pearson’s coefficient 0.485 ± 0.01; Fig. 4d and Table 3); however, the cause for this relatively high overlap appears to the higher background level of non-vesicle-associated dye (which is also observed in the dye-only control (Fig. 4f, left panel)). This conclusion is also supported by the high RWC1 (0.788 ± 0.03; suggesting that where there is dye there are likely to be EVs) but low RWC2 (0.073 ± 0.01; suggesting that where there are EVs there is little or no dye; Table 3). These data further confirm the poor labelling of vesicles and suggest that EV uptake experiments in which the EV-labelling dyes are imaged as a proxy for EV localisation should be interpreted with caution.

**Table 3:** A table summarising the quantitative colocalisation data of dual labelled mEmerald-CD81 EVs in recipient cells.

Stain used	Pearson’s coefficient	RWC1 (mEmerald-CD81 overlapping with stain)	RWC2 (stain overlapping with mEmerald-CD81)
Maleimide C5 AF633	0.212 ± 0.027	0.096 ± 0.032	0.261 ± 0.06
PKH26	0.485 ± 0.013	0.788 ± 0.034	0.073 ± 0.014

Data was acquired from image analysis using the JACOPx ImageJ plugin. Data was generated by analysing 30 individual cells per condition. N = 3, (Biological) ± SEM.

## Discussion

Caveats with lipophilic dyes have been well documented (reviewed in^36^), but maleimide and PKH26 have been used as a robust EV labels for dynamic EV tracking^19^. These dyes are commonly used in EV uptake and biodistribution studies. Here, we observed that only approximately 5% of fluorescent EVs were dual labelled with PKH26 or maleimide, highlighting how further caution should be used when utilising and extrapolating data with these dyes, and how there is an increased need for sophisticated approaches towards labelling bona fide EVs.

Recent studies have revealed several shortfalls of lipophilic dyes, including micelle formation, dye dissociation and non-specific staining^20,21,35^. Consistent with these studies, we observed non-specific aggregates produced by PKH26 and maleimide no EV controls. In both cases, we found that these aggregates were removed with SEC, suggesting, like others^20^, that with appropriate method optimisation (using particle free reagents/buffers or pre-clearing all reagents/dyes via ultracentrifugation prior to use) the proportion of contaminating dye could be decreased. Despite removing traces of free dye aggregates using SEC, we observed a low proportion of co-labelled EVs.

The ‘dye-positive, mEmerald-negative’ signals could be formed by non-specific dye interactions. Maleimide is known to bind to thiol groups present in proteins, and so it could feasibly bind to any free protein in the preparation. Recent evidence has revealed certain EVs can have different sized protein corona surrounding their membrane^37^. The Exoview sizing data revealed that maleimide-positive EVs were larger than GFP-positive EVs; this could suggest that maleimide binds to a different, larger subpopulation of EVs, or could reflect a change in the biophysical characteristics due to the presence of the dye. Alternatively, PKH26 has previously been shown to bind to lipoproteins^35^ and albumin^38^, which are known to be major contaminants of serum-containing medium. Takov et al.^35^ also observed more dye uptake in protein-rich EV samples, suggesting further non-specific binding. Therefore, when using these dyes there is an uncertainty whether the fluorescence observed is associated with an EV, lipoprotein, protein, or self-assembled dye.

Another contaminating factor contributing to the dye-positive mEmerald-negative particles, could be the presence of bovine EVs from the FBS present in the cell culture medium. Despite performing a preclearing FBS ultracentrifugation step to remove bovine EVs, it is possible that a small proportion of non-fluorescent bovine EVs remain, and these may be labelled by the EV dyes and contribute towards background in the colocalisation studies. Indeed, our nanoFCM data revealed that around half of all particles were not fluorescent in mEmerald-CD81 MCF7 EV preparations (either because they were non-vesicular particles, or because they did not contain mEmerald-CD81). This result was in line with previous reports assessing the amount of fluorescent particles after 72 h conditioning^27^. When we removed serum from our EV preparations this improved the PKH26 staining of EVs from ca. 5% to ca. 10%, and maleimide from ca. 2.5% to ca. 5.5%. This suggests that in both EV staining methods, a small amount of dye-positive mEmerald-CD81 EV-negative particles was due to the presence of serum components. However, even taking this bovine or MCF7 non-fluorescent EV contamination into account, the proportion of dual labelled EVs was concerningly low using commonly employed staining methods.

Conversely, we also had evidence of mEmerald-positive, dye-negative particles. A recent study investigating the robustness of conjugated tetraspanins for fluorescent labelling reported cleavage of the fluorescent protein from various constructs^27^, allowing the possibility that the mEmerald signals we observed could be not EV-associated. Here in this study, we confirmed that the GFP signals were membrane protected by using a proteinase K and Triton X-100 assay, suggesting that even if mEmerald cleavage occurs, the mEmerald signal is intraluminal and will reflect bona fide EVs. Furthermore, our Exoview analysis confirmed that GFP + particles were bound to each immunocapture spot suggesting specific fluorescent tagging.

The heterogeneity of EVs may also partially account for the lack of dual labelled mEmerald-CD81 EVs observed in this study. EVs contain a subset of cargo similar to their donor cell, however even EVs from the same cell line will have considerable heterogeneity leading to distinct subpopulations of EVs^13^. Individual EVs will originate from different biogenesis pathways, and although not fully characterised, it is understood that not all EVs subtypes will have the same cargo^39^, lipid profile or surface markers^40^ allowing for each EV to have different biophysical properties, and perhaps different interactions with the stains used. Jeppesen et al.^40^ suggested the presence of non-classical exosomes that were negative for the classical tetraspanin markers (CD9/CD63 and CD81). These non-classical EVs could be CD81-negative and contribute towards the ‘mEmerald-CD81-negative, dye-positive’ background seen in the dual labelled EVs in this study. Furthermore, there is increasing evidence suggesting that EV subpopulations positive for different markers do not behave in the same way; for example CD9- and CD63-positive HeLa EV subpopulations are internalised and trafficked through different routes, which perhaps is due to their different methods of biogenesis^41^.

Despite the particle size resolution of our confocal microscopy analysis being insufficient to confidently analyse single EVs, the 50 nm accuracy of ExoView Technology suggests the likely detection of individual EVs in our assays. Future experiments using alternative technologies such as Total Internal Reflection fluorescence (TIRF) microscopy^42^, fluorescence based NTA^43^ and other single-particle interferometric reflectance imaging sensing technologies (of which, Exoview is an example)^44^, could be useful to further validate our conclusion.

There is little information regarding confirming EV uptake using fluorescent dyes in the MISEV2018 guidelines^33^, other than ‘use careful interpretation’, and the use of appropriate controls. Reports have suggested that even with such controls, there is a concerningly high background level of uptake from these dyes alone without EVs. Lai et al.^21^ observed a 2.5-fold higher fluorescent signal from PKH67 stained non-conditioned medium than PKH67 stained EVs, confirming that caution should be used when interpreting EV uptake using these lipophilic dyes. We also recommend the use of density-based methods for removal of non-EV-associated dye, as such dye aggregates are likely to have a different density to bona fide EVs.

EVs are understood to be internalised by several endocytosis pathways and hemi-fusion routes^45^. There is evidence to show some types of EV uptake are preferred e.g. macropinocytosis^22,46^; however selective inhibition studies, using pharmacological or RNAi inhibition, have shown considerable redundancy in the uptake pathways used. Preferred uptake mechanisms may be donor/recipient cell-specific or dependent on EV characteristics. In short, we do not fully understand the complexities of EV uptake, and the molecular components involved. Progress in this area is somewhat limited by the lack of specific ways to definitively and accurately label EVs and accurately measure uptake. This study has revealed how some of the standard methods of tagging and visualising EVs may need standardisation and further optimising. It remains to be seen whether EVs labelled with other fluorescent tags, from other cell lines, or with other labelling methods also exhibit low labelling efficiencies. We recommend that researchers check the efficiency of their staining (particularly when describing new dyes and methodologies) before proceeding to experiments with stained EVs, and we suggest that should be a drive towards more reliable assays to assess EV uptake.

To conclude, this study highlights the low proportion of dual labelled EVs, when using PKH26 and maleimide. These labelling techniques have been important for furthering our understanding EVs, but we are suggesting that further optimisation is required to maximise their potential and that caution should be employed when interpreting the results of such experiments.

## Materials and methods

### Cell culture

HeLa cells (ATCC) and MCF7 cells (a kind gift from Munira Kadhim, Oxford Brookes University) were grown in Dulbecco’s Modified Eagle Medium (DMEM)/ Ham's F-12 Nutrient Mixture (HAM F12) medium plus 10% (v/v) Foetal Bovine Serum (FBS). MCF7 cells were transfected with an mEmerald-CD81 construct (mEmerald-CD81-10 Plasmid Addgene number 54031) and stably expressing colonies were selected using 600 µg/mL geneticin (G418) containing medium. The homogeneity of the mEmerald-CD81 positive MCF cell line was routinely checked by fluorescent microscopy (Primovert Cell and Tissue Culture Microscope; Zeiss) and flow cytometry (CytoFlex S flow cytometer; Beckman Coulter).

### Preparation of conditioned media for EV extraction

Cells were seeded at ~ 2 × 10^6^ MCF7 mEmerald-CD81-cells per T175 flask overnight. The cells were washed with PBS, medium was replaced with 25 mL DMEM F12 medium containing 5% (v/v) pre-cleared FBS (FBS previously centrifuged at 120,000×*g* for 16 h to pellet excess extracellular vesicles) and cells were cultured for 72 h. Conditioned medium was cleared by centrifugation at 300×*g* for 5 min followed by 16,500×*g* for 20 min at 4 °C. Approximately 50 mL of conditioned medium from two T175 flasks (approximately 90% confluent on collection), was concentrated using Vivaspin 20, 100 kDa (GE Healthcare) to 500 µL and used to isolate extracellular vesicles by size exclusion chromatography (SEC). Concentrated conditioned medium was either used immediately or frozen at − 80 °C until use.

Serum-free conditioned medium was made by seeding 4 × 10^6^ MCF7 mEmerald-CD81-cells per T175 flask overnight. The cells were washed with PBS and medium replaced with 25 mL serum free DMEM F12/HAM medium for 48 h, to condition. The conditioned medium was then prepared as above.

### Extracellular vesicle isolation by size exclusion chromatography (SEC)

Size exclusion columns were made using sepharose CL-2B (GE Healthcare). Briefly, 14 mL sepharose was added to an empty Econo-Pac chromatography Column (BioRad) and left to settle; a support bed was placed on top of column before washing twice with PBS. The 500 µL of concentrated medium sample was added and allowed to settle into the column, 10 mL of PBS was added, and 500 µL fractions were collected using PBS from the chromatography column. EV fractions (6 to 9) were kept, pooled and further concentrated using a Vivaspin 2, 5 kDa (GE Healthcare) to 200–400 µL for downstream experiments.

### Nanoparticle tracking analysis (NTA)

Nanoparticle tracking analysis was performed using Zetaview (Particle Metrix). EV samples and stained EVs were diluted in PBS (usually 1 in 1000–10,000 dilution) and were analysed for size and concentration using NTA video capture for 0.5 s at 11 positions at room temperature. The acquisition conditions were set as follows: Sensitivity = 80, Shutter speed = 100, Frame rate = 30, Cycle number = 2, Minimum brightness = 25, Maximum size = 1000, Minimum size = 5, Trace length = 15. The videos were analysed using ZetaView software version 8.04.12.

### Immunoblotting

EVs were concentrated in Vivaspin 2, 5 kDa, concentrator columns to 20 µL and lysed using 10X RIPA lysis buffer containing protease inhibitor cocktail III (1:100; Thermo Fisher). Lysates from cells or EVs (20 µg) were loaded into a 4–12% Bis–Tris NUPAGE gel (Invitrogen) either under reducing (with DTT; GM130) or non-reducing (Alix, TSG101, CD9 and CD81) conditions and run at 125 V for 90 min in NUPAGE MES SDS buffer. Gels were subject to a wet transfer onto a nitrocellulose membrane using NUPAGE transfer buffer (15% methanol) at 150 V for 2 h at 4 °C. Membranes were blocked in 5% (w/v) milk in 0.1% (v/v) PBST (PBS Tween-20) for 1 h. Membranes were probed with antibodies against: TSG101 (Abcam; ab83, mouse, 1:500) Alix (Abcam; Ab117600, mouse, 1:2500), CD9 (Cambridge Biosciences / System Biosciences; EXOAB-CD9A-1; rabbit 1:1000), CD81 (Abcam; ab79559, rabbit, 1:1000), and GM130 (Abcam; ab52649, rabbit, 1:1000). The membranes were then washed three times with PBST then probed in milk buffer with either anti-Rabbit conjugated horseradish peroxidase (HRP) (Promega; w4011, 1:5,000) or anti-mouse HRP conjugated secondary antibody (Promega; w402B, 1:20,000). The membranes were washed again three times with PBST, then developed using ECL reagent (Amersham; Pico) for 5 min (RT) and imaged using a Bio-Rad Gel Doc XR +.

### MACsPlex exosome immunocapture assay

The MACsPlex exosome immunocapture assay was carried out using a modified microcentrifuge tube protocol^47^. Briefly, 1 × 10^9^ EVs were incubated with 15 µL MACsPlex capture beads (Miltenyi Biotech) overnight rotating at 12 RPM. Beads were then washed in MACsPlex buffer and incubated with a cocktail of CD81-APC, CD63-APC and CD9-APC conjugated antibodies (Miltenyi Biotech) for 1 h. Beads were then washed twice with MACsPlex Buffer, resuspended in MACsPlex buffer, then fluorescence of the 39 bead populations were assessed by flow cytometry using a CytoFlex S flow cytometer (Beckman Coulter). Capture bead populations were resolved using channels B585 (PE) and B525 (FITC). Data was analysed by assessing the median fluorescence intensity (MFI) for APC (R660) for each gated bead population. Background bead MFI levels were subtracted from sample values and values were made relative to the average MFI for CD63/CD81/CD9 bead populations in MCF7 cells.

### Transmission electron microscopy

EV samples (10 µL) were pipetted onto carbon 300 mesh copper grids (TAAB, C267), previously glow discharged for 20 s at 15 mA, and incubated for 2 min. Grids were then dabbed against filter paper and placed onto a 20 µL drop of 2% (w/v) uranyl acetate for 10 s, then left to air-dry before storage. Grids were visualised using Jeol JEM-1400 Flash transmission electron microscope (TEM) with a Gatan OneView 16 Megapixel camera at 100 kV.

### EV dual-labelling with C5-maleimide-Alexa633

MCF7 mEmerald-CD81 EVs were stained with C5-maleimide-Alexa633 using a previously published protocol^19^. Briefly, this involved performing a BCA assay on un-lysed EVs and staining 60–100 µg EVs (total) in 50 µL PBS with 2.5 µL of 200 µg/mL C5—maleimide-Alexa633 (ThermoFisher) for 1 h, at room temperature, in the dark, with no agitation. The excess dye was removed using Exospin 3 kDa columns (Invitrogen) or by a making the sample up to 500 µL with PBS and performing an additional round of SEC.

### EV dual-labelling with PKH26

MCF7 mEmerald-CD81 EVs were stained with 2 µM PKH26 (Sigma). 20 µL EVs in PBS were diluted with 80 µL diluent C; 1 µL of PKH26 (final concentration: 2 µM) was added and the EVs were incubated in the dark at room temperature for 10 min. The excess dye was removed using Exospin 3 kDa columns (Invitrogen) or by a making the sample up to 500 µL with PBS and performing an additional round of SEC.

### Proteinase K and Triton X-100 treatment of EVs

Concentrated MCF7 CD81emGFP EVs were treated with 100 µg/mL Proteinase K (Invitrogen) in isolation or with 1% (v/v) Triton X-100 (Sigma) then incubated at 37 °C for 1 h, with vortexing every 15 min. After treatment(s), EVs were placed under coverslips for confocal imaging.

### Exoview experiments

Exoview R100 (Nanoview BioSciences, UK) experiments were performed at Nanoview BioSciences UK facilities according to their protocols. Briefly, EVs were prepared and stained as above from conditioned media containing 10% (v/v) pre-cleared FBS. 20 µL of diluted EVs were incubated overnight on Exoview chips containing immunocapture spots for CD81, CD63, CD9 and MIgG, in triplicate. Exoview chips were washed three times with incubation solution then stained with antiCD81-AF555 antibody for 1 h. Exoview chips were washed three times, then imaged using the Exoview R100 and analysed for fluorescence and size using the ExoScan 2.5.5 acquisition software (NanoView Biosciences, UK).

### Preparation of EVs in 1% agarose for confocal imaging

EVs were mixed with 2% (w/v) low melting agarose in TAE buffer (cooled to just above melting temperature) in a 1:1 ratio (10 µL:10 µL), then the mixture (20 µL) was pipetted onto a glass microscope slide with a 13 mm diameter cover slip placed on top. This was left to dry, then secured by clear nail polish.

### Uptake assay

HeLa cells were seeded overnight in 8-well chamber slides with removable wells (Nunc Lab-TEK II Chamber slide system; Thermo Fisher) at a density of 20,000 cells/ well. Cells were treated with dual stained (mEmerald-CD81 EVs stained with either PKH26 or Maleimide) or unstained mEmerald-CD81 EVs for 3 h. The dose of EVs was equivalent to 50 × the growth area of the cells (e.g. EVs harvested from a T175 flask used to treat one well [growth area 1 cm^2^] would equal a 175 × dose). After EV treatment cells were optionally stained with cell mask orange or cell mask deep red (Thermo Fisher), washed with PBS and fixed in 4% (w/v) Paraformaldehyde for 15–20 min at room temperature. Fixed cells were then washed three times with ice cold PBS, incubated with 30 mM glycine for 5 min and washed before aspirating the buffer, removing the wells and mounting a coverslip on top with DAPI containing Prolong gold antifade mounting agent (Thermo Fisher).

### Confocal imaging

EVs were imaged using a Zeiss LSM880 or Zeiss LSM800, as indicated on individual figure legends. Images of EVs in agarose were taken using a 100 × objective (LSM800; 100x = NA 1.4, LSM880; 100x = NA 1.46). Images of cells were captured using a 63 × objective (NA = 1.4). Images were acquired using 4 × line averaging. Z stacks were taken sectioning the height of the agarose/ cells.

### Image analysis

Images were analysed using Zen black software (Zeiss) or ImageJ (FIJI)^48^. Colocalisation of dual labelled EVs was analysed through the JACOPx Plugin on ImageJ FIJI^49^. Pearson’s coefficient, Rank-weighted colocalisation (RWC)^49^, fluorescent particle counts and colocalisation based on centres of mass-particles coincidence were performed using the JACOPx Plugin settings with manually adjusted thresholds, matching the intensity of particles seen on the software with the original image captured. The percentage of dual stained EVs was calculated using the object based ‘colocalization based on mass-particle coincidence’ feature of the JACOPx plugin. This automatically quantified the number of particles and assessed overlap of pixels calculated between the channels. The values of coincidence were then used to calculate the percentage of dual stained EVs as a proportion of the individual EV particles counted.

### Statistical analysis

Statistical analysis was performed on GraphPad Prism using 2-way ANOVAs and Student’s unpaired t-test, as stated in figure legends. All error bars are standard error mean (SEM) unless otherwise stated. All graphical figures were prepared using GraphPad Prism.

## Supplementary Information

Supplementary Information 1.

Supplementary Information 2.

## Abbreviations

EV: Extracellular vesicles
SEC: Size exclusion chromatography
NTA: Nanoparticle tracking analysis
RWC: Rank-weighted colocalisation
MVB: Multivesicular body
