# Supplementary material for: Confocal microscopy analysis reveals that only a small proportion of extracellular vesicles are successfully labelled with commonly utilised staining methods
Source: Sci Rep. 2022 Jan 7;12:262. doi: 10.1038/s41598-021-04225-4 (PMC8741769; doi:10.1038/s41598-021-04225-4)
Supplement: Supplementary file 1 [file 41598_2021_4225_MOESM1_ESM.odt]

Supplementary figure legends 

Supplementary Figure 1: NanoFlow cytometry revealed over half the particles in mEmerald-CD81 EV samples are fluorescent. MCF7 EVs (top row) and mEmerald-CD81 MCF7 EVs (bottom row) were analysed by nanoFCM to assess their size and fluorescence. Histograms from the NanoFCM showing the gating by side scatter (left column) and fluorescence (right column). The gating strategy was set using background fluorescence (MCF7 EV), anything above this was deemed fluorescent. Left column was gated for fluorescence, the right column for size (size was gated for according to the size profile of typical EV preparations).

Supplementary Figure 2: Colocalisation was not improved by using SEC rather than exosome columns. Confocal images of dual stained EVs processed using either Exosome columns or SEC were analysed using the JACOPx ImageJ plugin for colocalisation using Pearson’s coefficient (A), RWC (B) and % Dual Stained EVs (using colocalisation based on centres of mass-particles coincidence. C). Statistical analysis 2-way ANOVA demonstrated no statistical significance between any comparisons.  N=3 (biological) ±SEM.

Supplementary Figure 3: Exoview analysis showed low percentage maleimide colocalization in CD81+ EV subpopulations. Exoview analysis of Maleimide stained mEmerald-CD81 MCF7 EVs bound to the CD81 immunocapture spot were analysed for Maleimide, GFP and conjugated antiCD81-AF555 fluorescence. CD81-PE positive particles on the considered CD81+ EVs and analysed for the presence of Maleimide and or GFP (mEmerald). % colocalisation for the CD81+ EV population was plotted (A). B shows a representative image of the CD81 immunocapture spot, white circles indicate any colocalization between antiCD81-AF555 (green), Maleimide (red) and GFP (blue). N=3 (biological) ±SEM.

Supplementary Figure 4: Colocalisation of Maleimide with mEmeraldCD81 MCF7 EVs was low across CD81+, CD63+ and CD9+ subpopulations of EVs.  Exoview analysis of Maleimide stained mEmerald-CD81 MCF7 EVs bound to immunocapture spots CD9, CD63, CD81 and MIgG were analysed for Maleimide, GFP and antiCD81-AF555. Total number of particles counts for CD81+, GFP+, Mal+, CD81+GFP+, CD81+Mal+, GFP+Mal+ and CD81+GFP+Mal+ EV populations are shown for the CD81 (A), CDCD63 (B) CD9 (C) and MIgG (D) immunocapture spots separately, and a representative image of the immunocapture spot used for automated analysis. AntiCD81-AF555 (green), Maleimide (red) and GFP (blue). N=3 (biological) ±SEM.

Supplementary Figure 5: Maleimide positive particles were significantly larger than GFP positive particles. Exoview analysis of Maleimide stained mEmerald-CD81 MCF7 EVs bound to Immunocapture spots CD9, CD63, and CD81 were analysed for size and fluorescence. The average median sizes of particles that were positive for Maleimide staining and negative for GFP were compared to the average median sizes of particles that were positive for GFP and negative for Maleimide staining. **p= 0.0097 (CD63), ***p=0.0001 (CD9) and ****p=<0.0001 (CD81), using an individual unpaired t-test with Welch’s correction for unequal variance. N=3 (biological) ±SEM.

Supplementary Figure 6: Removing serum from EV preparations increases staining efficacy. Composite confocal images of mEmerald-CD81 MCF7 EVs prepared from serum free conditioned media, dual stained with Maleimide C5 AF633 (a) or PKH26 (b), excess dye removed with Exospin columns, then set in 1% agarose. Scale bar is 10 µm. 

Supplementary Table 1: A table summarising the Exoview sizing data comparing Maleimide positive and GFP positive particles. Exoview (Nanoview) analysis of Maleimide stained mEmerald-CD81 MCF7 EVs bound to Immunocapture spots CD9, CD63, CD81 and MIgG were analysed for size and fluorescence. The average median sizes of particles that were positive for Maleimide staining and negative for GFP were compared to the average median sizes of particles that were positive for GFP and negative for Maleimide staining. **p= 0.0097 (CD63), ***p=0.0001 (CD9) and ****p=<0.0001 (CD81), using an individual unpaired t-test with welch’s correction for unequal variance. N=3 (biological) ±SEM.

Supplementary Table 2: A table summarising the quantitative colocalization of mEmerald-CD81 MCF7 EVs, from serum free preparations, dual stained with Maleimide C5 AF633 or PKH26. The data was acquired from image analysis using the JACOPx ImageJ plugin. N=3 (Biological) ±SEM. *p≤0.05, **p≤0.001 assessed by two tailed, unpaired Student’s t-test compared to serum conditioned medium extracted EVs prepared in the same way.

Supplementary figures

A screenshot of a computer screen

Description automatically generated

Supplementary Figure 2: Colocalisation was not improved by using SEC rather than exosome columns.

a

c

b

a

Supplementary Figure 3: Exoview analysis showed a low percentage of mEmeraldCD81 MCF7 EVs were colocalised with Maleimide.

b

 

Supplementary Figure 4: Colocalisation of Maleimide with mEmeraldCD81 MCF7 EVs was low across CD81+, CD63+ and CD9+ subpopulations of EVs.  

a

b

c

d

Supplementary Figure 5: Maleimide positive particles were significantly larger than GFP positive particles. 

Supplementary figure 6: Removing serum from EVs preparations increases staining efficacy with PKH26 and Maleimide

a

b

Supplementary table 1: A table summarising the average median sizes between Maleimide positive particles and GFP positive particles

| Immunocapture spot | Median size of Maleimide positive particles | Median size of GFP positive particles |
| --- | --- | --- |
| CD9 | 63.23 +/- 0.22 | 56.24 +/- 0.32 *** |
| CD63 | 61.70 +/- 0.92 | 55.92 +/- 0.57 ** |
| CD81 | 62.34 +/- 0.24 | 55.58 +/- 0.20 **** |
| MIgG | 56.38 +/- 32.27 | 43.19 +/- 43.19 |

Supplementary table 2: A table summarising the quantitative colocalization of mEmerald-CD81 MCF7 EVs, from serum free preparations, dual stained with Maleimide C5 AF633 or PKH26.

| Stain Used | Pearson’s coefficient | RWC2 (stain overlapping with mEmerald-CD81) | % Dual stained/ colocalization based on centres of mass-particles coincidence |
| --- | --- | --- | --- |
| Maleimide C5 AF633 | 0.162 ± 0.055 | 0.143 ± 0.043 | 5.55% ± 0.68% * |
| PKH26 | 0.126 ± 0.011 * | 0.094 ± 0.056 | 10.97% ± 0.49% ** |
