# Supplementary material for: Confocal microscopy analysis reveals that only a small proportion of extracellular vesicles are successfully labelled with commonly utilised staining methods
Source: Sci Rep. 2022 Jan 7;12:262. doi: 10.1038/s41598-021-04225-4 (PMC8741769; doi:10.1038/s41598-021-04225-4)
Supplement: Supplementary file 2 [file 41598_2021_4225_MOESM2_ESM.odt]

Uncropped blots from figures 

Fig 1d

Membranes were cut at appropriate sizes to maximise sample usage. The red boxes show the lanes used to make the figure. 

HSP70 

TSG101 (Ab63) 49kDA

CD9 26kDa

GM130

Beta Actin

Cytochrome c

Anti-CD81

Fig 1e

Anti-β-actin (on right, left is other samples)
